# Characterization of the thermolysis products of Nafion membrane: A potential source of perfluorinated compounds in the environment

**DOI:** 10.1038/srep09859

**Published:** 2015-05-07

**Authors:** Mingbao Feng, Ruijuan Qu, Zhongbo Wei, Liansheng Wang, Ping Sun, Zunyao Wang

**Affiliations:** 1 State Key Laboratory of Pollution Control and Resources Reuse, School of the Environment, Nanjing University, Nanjing 210023, P. R. China

## Abstract

The thermal decomposition of Nafion N117 membrane, a typical perfluorosulfonic acid membrane that is widely used in various chemical technologies, was investigated in this study. Structural identification of thermolysis products in water and methanol was performed using liquid chromatography-electrospray ionization-tandem mass spectrometry (LC/ESI-MS/MS). The fluoride release was studied using an ion-chromatography system, and the membrane thermal stability was characterized by thermogravimetric analysis. Notably, several types of perfluorinated compounds (PFCs) including perfluorocarboxylic acids were detected and identified. Based on these data, a thermolysis mechanism was proposed involving cleavage of both the polymer backbone and its side chains by attack of radical species. This is the first systematic report on the thermolysis products of Nafion by simulating its high-temperature operation and disposal process via incineration. The results of this study indicate that Nafion is a potential environmental source of PFCs, which have attracted growing interest and concern in recent years. Additionally, this study provides an analytical justification of the LC/ESI-MS/MS method for characterizing the degradation products of polymer electrolyte membranes. These identifications can substantially facilitate an understanding of their decomposition mechanisms and offer insight into the proper utilization and effective management on these membranes.

In recent decades, polymer electrolyte membranes (PEMs) have played an increasingly important role in numerous areas of chemical, biological and engineering applications, such as fuel cells, electrodialyzers and sensors^1^,^2^. In particular, PEM fuel cells, a promising zero-emission power source, have been extensively investigated owing to their great potential in supplying energy to automobiles, for stationary power generation and in portable electronic devices^3^. Previous research has shown that cell performance and lifetime were closely related to the characteristics of PEMs as observed under operating conditions^4^,^5^,^6^. As a typical perfluorosulfonic acid (PFSA) membrane, Nafion consists of a polytetrafluoroethylene (PTFE) backbone with perfluoroalkylether pendant chains terminating in sulfonic acid groups. Nafion has been widely employed in fuel cells because of its high proton conductivity, good chemical stability and high mechanical strength^1^,^7^,^8^. Recently, studies have primarily focused on its chemical degradation in fuel cells^7^,^9^,^10^,^11^, its utilization in modified electrodes^12^,^13^, and its use in the removal of environmental pollutants^14^,^15^. In particular, the chemical degradation of Nafion in PEM fuel cells can largely deteriorate cell performance and durability with decomposition mechanisms consistently attributed to the chemical attack by trace radical species such as hydroxyl radicals^9^,^10^,^11^.

In view of the increasing use of Nafion in various chemical technologies, it is important to establish waste treatment techniques for this PEM. As suggested by the manufacturer (DuPont), incineration is one of the current approaches for Nafion disposal^16^. According to the “trace chemistries of fire” hypothesis proposed by Crummett and Townsend^17^, almost all processes of combustion are incomplete and can cause the formation of a broad set of chemical products. This viewpoint can also be applied to thermolysis processes, of which numerous products are commonly generated and are highly influenced by operating conditions, such as oxygen supply and temperature programming^18^. Several studies have shown that the thermolysis of PTFE fluoropolymer can produce a variety of perfluorinated compounds (PFCs), such as environmentally persistent perfluorocarboxylic acids (PFCAs)^19^,^20^. These chemicals have increasingly attracted worldwide concerns due to their persistence in environmental matrices and potential negative impacts on living organisms^21^,^22^,^23^,^24^. Given the PTFE backbone in Nafion, it is reasonable to assume that some similar products can also be generated when it is thermally treated. Liquid chromatography/electrospray ionization tandem mass spectrometry (LC/ESI-MS/MS) has been recognized as a powerful analytical tool for the precise identification of multiple components. In general, this method combines the high chromatographic resolution of LC with the high specificity and sensitivity of MS/MS^25^,^26^. Notably, this method has been used to characterize the decomposition products of a specific PEM in fuel cell water^27^,^28^ and to identify perfluorooctanoic acid (PFOA), a PFCA analogue of vital importance, released from commercial cookware under operating conditions^29^,^30^. Unfortunately, little is known regarding the compositional analysis and structural identification of Nafion thermolysis products using this method.

In the present work, we report on the thermolysis products of Nafion N117 (Fig. 1), a typical PFSA membrane, absorbed in water and methanol using a LC/ESI-MS/MS screening method. The release of F^−^ ions during PEM thermolysis was studied with an ion-chromatography system. Additionally, thermal stability was characterized by thermogravametric analysis (TGA). The objectives of this study were to investigate the possible production of environmentally significant PFCs generated via N117 thermolysis and to propose thermal degradation mechanisms based on key products observed in previous studies and additional products detected in this work. To the best of our knowledge, this study is the first to report not only on the application of a LC/ESI-MS/MS method to characterize the thermolysis products of Nafion membrane but also on the potential impacts of its incineration or high-temperature operation. It is also noteworthy that the latter could potentially cause the release of PFC species that could contribute to environmental pollution and deterioration of membrane performance.

## Results

### Thermogravametric analysis

The TGA curve of N117 following increasing temperatures is shown in Fig. 2 and is accompanied by enhanced presentations of certain transitions. Three main stages were observed with their temperature ranges recorded at 75–200°C, 275–420°C, and 420–600°C.

### Characterization of the thermolysis products of N117

The total ion current (TIC) chromatogram of NaOH absorption solution is illustrated in Fig. 3a. The peak assignments of some products are listed in Table 1 with their proposed structures, retention times, signal intensity and MS characteristics. As shown in Fig. 3a, there were many chromatographic peaks in the TIC. Specifically, deviations of 50n (n = 1, 2, 3, …) between the *m/z* values of certain products correspond to the “CF_2_” unit. After peak classification, four groups of chemical analogues were characterized in the NaOH absorption solution.

Group 1, represented by *m/z* 112.9870 and 412.9658, was tentatively identified as the PFCA analogues with the general structure of CF_3_(CF_2_)_n_COO^−^. This structure was proposed according to their major MS/MS fragments (68.9953 and 45.0005 for *m/z* 112.9870; 368.9759, 218.9851 and 168.9885 for *m/z* 412.9658), while the structures of some representative *m/z* values are shown in Table 2. To further validate the generation of PFCAs, several products were subjected to the MS/MS analysis and compared with their possible standards. As shown in Supplementary Fig. S1 online, good agreement was commonly observed between these products and their standards. Additionally, based on some representative *m/z* values, group 2 (*m/z* 394.9780, 544.9698), 3 (*m/z* 424.9921, 524.9636) and 4 (*m/z* 338.9692, 388.9697) were also detected and largely classified into three different chemical analogues. Their respective structures were ambiguously proposed as CF_3_(CF_2_)_n_CHFCOO^−^, CF_3_(CF_2_)_4_CF = CF(CF_2_)_n_COO^−^ and CF_3_(CF_2_)_n_CF(COOH)COO^−^ according to their major MS/MS fragments. In addition, these structures were further confirmed with minor discrepancies between the observed and calculated *m/z* values (Table 1).

Additionally, two deprotonated molecular [M-H]^−^ ions at *m/z* 96.9624 and 130.9619 corresponded to the structures of HSO_4_^−^ and CHF_2_SO_3_^−^. Their identifications were confirmed by comparison with the N117 structure and the observed MS/MS fragments (79.9598 and 79.9590, Table 2), corresponding to the loss of an OH unit [M-H^−^ 17]^−^ and a CHF_2_ unit [M-H^−^ 51]^−^, respectively. In addition, sulfate ions (SO_4_^2-^), together with F^−^ ions, were also detected in the IC (see Supplementary Fig. S2 online), with their individual retention times at 6.853 and 3.037 min. Further validation was performed by comparing these data with the IC peaks of NaF (20mg L^−1^) and Na_2_SO_4_ (20mg L^−1^).

The TIC chromatogram of N117 thermolysis products absorbed with methanol is shown in Fig. 3b, and the peak assignments of some products are presented in Table 3. Their possible structures were ambiguously proposed based on the MS/MS fragments (see Supplementary Table S1 online) of two representative *m/z* values for each group (236.9847, 286.9848 for group 4; 252.9848, 352.9721 for group 5; 472.9934, 522.9896 for group 6; 340.9925, 390.9877 for group 7; 158.9915, 208.9867 for group 8). The detailed interpretations on the MS/MS fragments of five representative PFC analogues and the other products are illustrated in Supplementary Fig. S3 and Fig. S4 online, respectively. As for the formation of PFCA products, different analogues in NaOH and methanol absorption solutions and extracted ion chromatograms (EIC) corresponding to the deprotonated molecular ion [M-H]^−^ of PFOA (*m/z* 412.97) are presented in Fig. 4. Notably, PFCAs with different chain lengths were commonly found, although their presence varied between the two different extraction solvents.

## Discussion

To become commercialized in various chemical applications, Nafion-related technologies such as PEM fuel cells must be able to function within a wide temperature range and under temperature cycling conditions^7^. Currently, a number of studies have been conducted to design fuel cells that can operate above 100°C to enhance electrochemical kinetics, simplify cooling systems, facilitate water management, and improve system CO tolerance^3^,^5^,^7^. However, operation of these fuel cells for an extended time at high temperatures can accelerate membrane degradation and inevitably leads to concerns regarding the thermal stability and response of the Nafion electrolyte. Furthermore, the waste disposal of Nafion components via incineration may produce undesirable thermal decomposition products. Thus, the current study was conducted to systematically investigate the thermolysis of Nafion membranes by TGA, LC/ESI-MS/MS and IC analysis.

Currently, TGA has been commonly utilized as an effective tool for studying the thermolysis of chemicals^6^,^31^,^32^. Specifically, the thermal stability of PFSA membranes (e.g., Nafion) has been previously studied by this method^6^,^33^,^34^. This study has consistently shown three stages of thermal degradation in Nafion. These stages correspond to the loss of water, the cleavage of C-S bond, and the decomposition of the perfluorinated matrix, respectively. In the present research, these three stages were also observed during N117 thermolysis. These results were largely in accordance with several previous reports^6^,^34^. A detailed analysis of the thermal decomposition process of N117 is presented in the following sections.

To better analyze the possible thermolysis products of N117, two different solvents were used in parallel for the products absorption. The two solvents were water [polarity index (*P*) = 9, dipole moment (DM) = 1.85], with NaOH dissolved in it, and methanol (*P* = 5.1, DM = 1.70), thus yielding two different absorption solutions. In a recent study, Kaur et al.^35^ adopted five different extraction solvents of various polarity or “extraction strengths” to initiate the chemical characterization of the combustion products of chlorogenic acid. Furthermore, several previous studies have also used water and/or methanol to extract PFOA from PTFE fluoropolymer^36^,^37^ and the surface of commercial cookware^30^. In our study, more thermolysis products were detected in methanol than those in NaOH solution, which may be attributed to the higher extraction strength of methanol.

Among these thermolysis products, several new bonds, such as C-H, C = O and C = C, were introduced into the proposed structures when compared to the structure of N117. Generally, these bonds have been reported to exist in the main chain of Nafion as undesired byproducts of the manufacturing process^38^,^39^. On the other hand, Danilczuk et al.^40^ reported the formation of C-H and C = O bonds during Nafion degradation in the fuel cell by 2D spectral-spatial FTIR. Additionally, the formation of C = C bonds has been suggested experimentally and theoretically in alkaline treated polyvinylidene fluoride (PVDF) membrane^41^,^42^. This may be equally applied to Nafion thermolysis given their structural similarity. Hydroxyl groups were partially incorporated into N117 thermolysis products, contributing to their structural diversity. In a previous study, hydroxyl groups were also observed in PVDF membranes treated with alkaline solutions^42^.

Notably, in this work, PFCA analogues of chain lengths ranging from C_1_ to C_18_ were readily observed during N117 thermolysis. In the recent past, PFCAs have been ubiquitously detected and characterized as persistent, bioaccumulative, and toxic (PBT) pollutants^21^,^22^,^23^,^24^. Thus, increasing attention has been focused on understanding the behavior and fate of these analogues in multiple environmental matrices. In an effort to reduce the environmental impact of PFCA analogues, systematic studies are required to elucidate their potential environmental sources. Previous studies have suggested that PFCA emission during manufacturing had the greatest environmental impact^43^,^44^. Meanwhile, the formation of PFCAs from some potential precursors such as fluorotelomer alcohols^45^, polyfluoroalkyl phosphates^46^, and polyfluorinated amides^47^, has also been confirmed. The current study suggests that the thermolysis process of N117 could also be a potential environmental source of PFCAs, as validated by comparing the MS/MS data against standards (see Supplementary Fig. S1 online). Specifically, trifluoroacetic acid (TFA) was detected as the major analogue. Additionally, the amount of long-chain PFCAs (n > 8) was found to decrease with the increasing chain lengths. These observations coincided well with the product composition of PTFE thermolysis^20^. TFA, one of the major pollutants in rainwater^48^, has also been observed in fuel cell degradation tests^49^ and Nafion degradation in subcritical water with zerovalent metals^8^. Notably, long-chain PFCAs have gained specific interest by some global regulatory communities^50^,^51^. They have been regarded as vPvB chemicals (very persistent and very bioaccumulative) due to their degradation resistance and high accumulation potentials^52^. Together with PFOA, they were included in the Candidate List of Substances of Very High Concern under the European chemical regulation, REACH^53^.

In addition to several groups of chemical analogues, CHF_2_SO_3_^−^ and SO_4_^2-^ were also observed as two single products during N117 thermolysis. Specifically, the formation of CHF_2_SO_3_^−^ was also found in Nafion degradation using zerovalent metals in subcritical water^8^, suggesting the possible linkage of its pendant chain. Previous research on Nafion degradation has also indicated the generation of SO_4_^2-^ and F^−^ ions whether under *in situ* (fuel cell operation) or *ex situ* (Fenton test) conditions^9^,^54^,^55^. Recently, Yu et al.^11^, using quantum mechanical calculations, proposed two mechanisms for OH^•^ attack on Nafion polymer that also involved the formation of these two ions. Notably, SO_4_^2-^ and F^−^ ions were observed by solution analysis of Nafion after being heated at 433 K for 12 h^54^, which is consistent with results from the current study on N117 thermal decomposition.

It is noteworthy to mention that in this study several different thermolysis products, especially the PFCs, were detected and identified after the solution analysis. These new-found substances may also be generated under the high-temperature conditions found during the operation of PEM fuel cells, and they have the potential to impact cell performance and lifetime. Regarding the researchers who focus on extending the cell durability, the possible effects induced by these products should be systematically evaluated and effectively avoided. Additionally, it is well established that PFCs, such as those produced during Nafion disposal, will significantly contribute to pollution on a global scale. In addition to the PFCAs, several previously unreported PFCs such as groups 3–5 were also observed as the thermolysis products of Nafion fluoropolymer. Moreover, some long-chain PFCs, which have been reported to be more bioaccumulative than short-chain analogues^21^,^56^, were also found in this investigation. Although most efforts thus far have focused on PFCAs, it is also of vital importance to assess and understand the environmental disposition and toxicity of some other PFCs.

Despite the increasing interest in elucidating the chemical degradation of Nafion (see Supplementary text online), a limited amount of research has been conducted to systematically understand the possible mechanisms of Nafion thermolysis. In this study, a mechanism was tentatively proposed as involving the cleavage of both polymer backbone and its side chains by attack of radical species. Samms et al.^34^ and Wilkie et al.^57^ proposed a thermal degradation mechanism of Nafion at higher temperatures that included an initial C-S bond cleavage to yield sulphur dioxide, OH^•^ radicals, and a carbon-based radical. In our work, a variety of thermolysis products of N117 were identified. Relevant to the PFCA analogues generated, Ellis et al.^19^ reported several pathways in the thermal decomposition of PTFE polymer that involved the formation of carbene radicals, longer-chain diradicals, and a series of reactions with other species. These proposed pathways may also be adapted to N117 thermolysis given the shared PTFE backbone. Alternately, C = C bonds in the products may form from an attack of OH^•^ radicals on the unintentionally introduced C-H defects present in the main chains (eq 1). 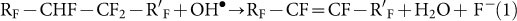 In addition, the common detection of HSO_4_^−^ and CHF_2_SO_3_^−^ in different absorption solutions indicated cleavage of the Nafion side chain. The formation of these species was also found accompanying chemical degradation^8^,^54^,^55^. In general, thermally produced radicals can react with the pendant chain under high temperatures, leading to the production of ^•^CF(CF_3_)OC_2_F_4_SO_3_^−^. This species has also been reported in Nafion degradation using zerovalent metals in subcritical water^8^. Subsequent to its production, this radical can be cleaved to generate CF_3_COF, and further hydrolysed to form TFA (eq 2-3). 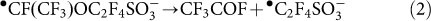


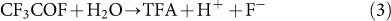


Recently, radical reactions involving the Nafion side chain have been theoretically confirmed by frontier orbital theory^55^. In this study, the calculated highest occupied molecular orbital (HOMO) and lowest unoccupied molecular orbital (LUMO) showed a wide distribution of the electron cloud around the side chain. This phenomenon may also indicate potential reaction sites preferred by radicals generated during N117 thermolysis. Although detailed mechanisms on the formation of some species, such as CHF_2_SO_3_^−^, remain unclear, the obtained results regarding these thermolysis products can help to elucidate a more accurate mechanism of Nafion thermal degradation. Recently, two investigations also utilized the LC/ESI-MS/MS method to determine the degradation products of sulfonated polyarylether membrane in fuel cell water^27^,^28^. Together with the current work, these studies collectively indicate the potential this method has for characterizing the products of PEM decomposition, which may be triggered by attack of radical species generated under realistic operating conditions.

## Conclusions

The thermal degradation of Nafion was investigated by mimicking the conditions of high-temperature operation typical of several chemical applications, and the waste disposal process via incineration. By analyzing two different absorption solutions, multiple groups of related thermal decomposition products were identified and their structures were proposed. These results indicated the structural diversity of these thermolysis products, which allowed for proposing thermal degradation mechanisms. With regards to technical applications, such as PEM fuel cells, these thermally generated Nafion products may potentially hinder cell performance and lifetime. With respect to the environment, the production of numerous PFCs, such as PFCAs, during Nafion disposal will contribute to pollution on a global scale. It is important to note that this study has identified a potential source of PFCs, which will continue as a class of emerging POPs representing a long-term challenge to scientists in the foreseeable future. Additionally, this study showed that the LC/ESI-MS/MS screening method is a powerful and effective tool for the detailed elucidation of the decomposition mechanisms of the current-use PEMs in chemical technologies.

## Methods

### Chemicals

**Figure 1:**
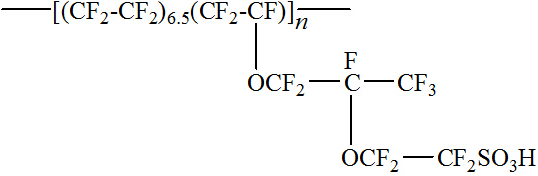
Chemical structure of Nafion N117 membrane.

**Figure 2:**
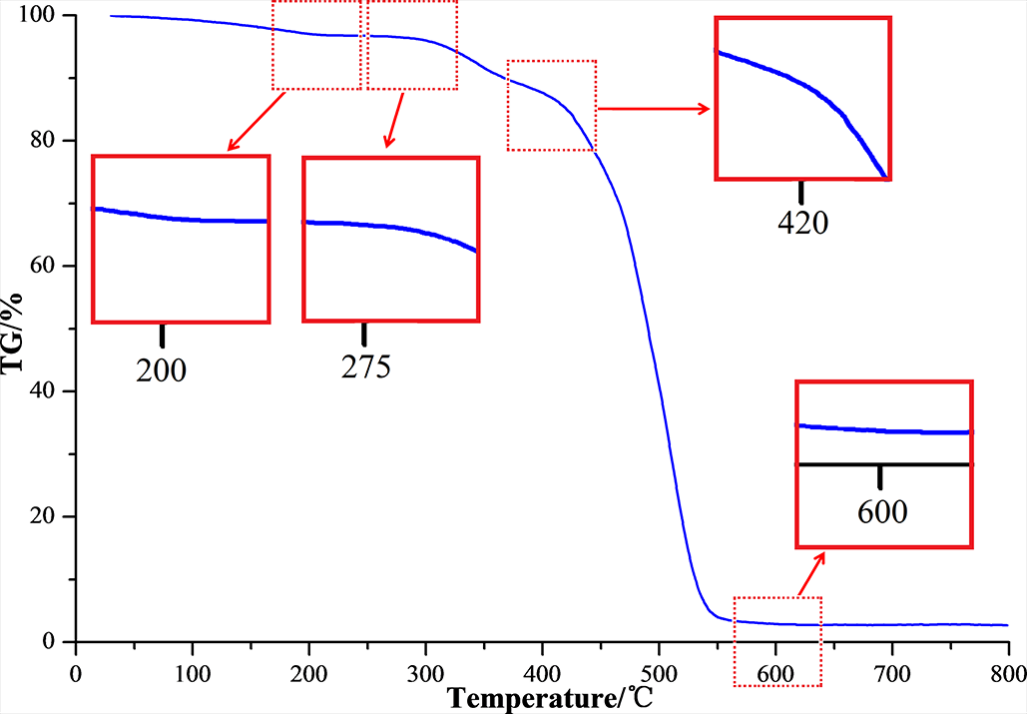
TGA curve of N117 membrane under nitrogen atmosphere (heating rate: 20°C min^-1^). Insets represent the zoom-ins of the areas in red dashed-line boxes.

**Figure 3:**
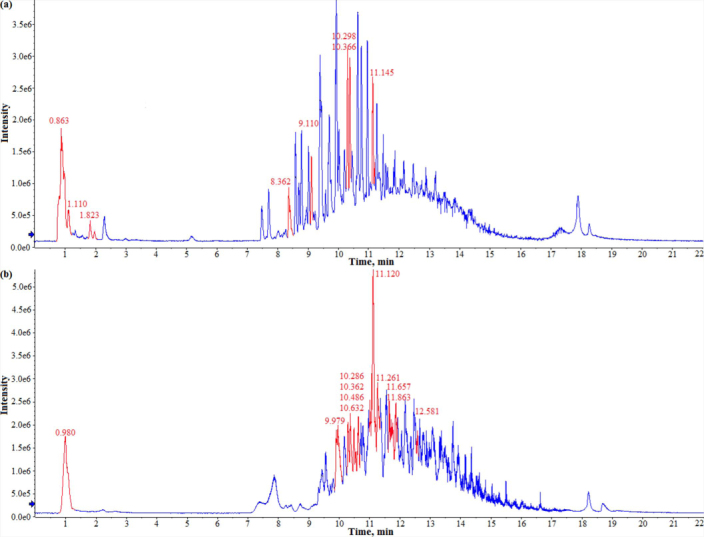
Total ion current (TIC) chromatograms of NaOH (a) and methanol (b) absorption solutions showing the various thermolysis products of N117, which were analyzed by LC/TOF-MS in a negative ion mode. The peaks of some representative *m/z* values listed in Table 1 and 3 were highlighted in red.

**Figure 4:**
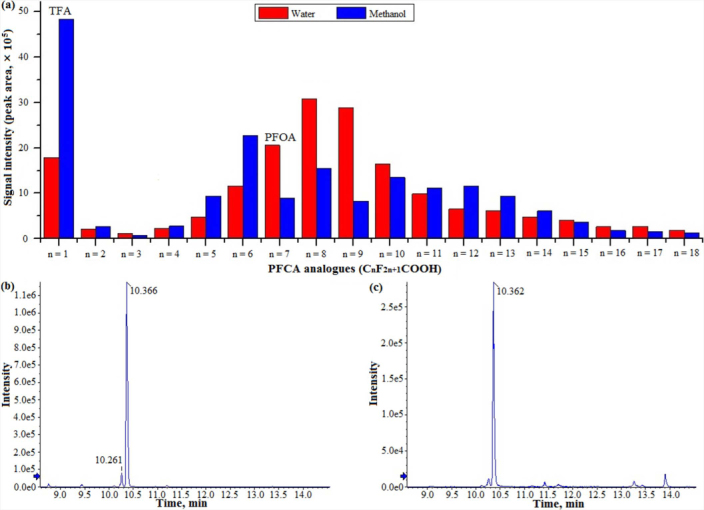
LC/ESI-MS/MS analysis of the PFCA analogues (C_n_F_2n+1_COOH, n = 1-18) in NaOH and methanol absorption solutions (a), and extracted ion chromatograms (EIC) corresponding to the deprotonated molecular ion [M-H]^−^ of PFOA (*m/z* 412.97) in NaOH (b) and methanol (c) absorption solutions.

Nafion N117 membranes (basic weight: 360g m^−2^, typical thickness: 183μm) were obtained from Shanghai Hesen Electric Co., Ltd (Shanghai, China). The chemical structure is presented in Fig. 1. No pretreatment was performed on this membrane prior to its use. The LC/MS grade solvents were used for the chromatographic separation (water and acetonitrile) and thermolytic absorption (methanol). Together with the additive formic acid, they were supplied from Merck (Darmstadt, Germany) and utilized without further purification. PFCA analogues (see Supplementary Table S2 online for their structures) were obtained from J&K Chemical Co., Ltd. (Shanghai, China). Sodium hydroxide (NaOH), sodium fluoride (NaF) and sodium sulfate (Na_2_SO_4_) were purchased from Nanjing Chemical Reagent Co., Ltd. (Nanjing, China). Aqueous solutions were prepared with ultrapure water (> 18.2MΩ cm), obtained from a Milli-Q Plus system (Millipore, Bedford, MA, USA).

### Thermogravimetric analysis (TGA)

The thermal stability of N117 was characterized using a Perkin-Elmer TGA 4000 instrument (Perkin-Elmer Inc., Wellesley, MA, USA). TGA test was conducted with a specimen weighting approximately 20mg at a constant heating rate of 20°C min^−1^ over a temperature range of 30–800°C under nitrogen atmosphere.

### Thermolysis procedure

Approximately 0.5g N117 pieces were heated in specialized pyrolysis equipment (AZ-HC-06a, Tianjin Aozhan Technology Co., Ltd, Tianjin, China). The operating parameters including the atmosphere, heating temperatures and running time were set according to the literature^16^ with a slight modification, while the related thermolysis data are cited in Supplementary Table S3 online. Specifically, the atmosphere was air, and flow rate was set as 13mL min^−1^. The N117 sample was heated in a stainless steel tube at 10°C min^−1^ to 200°C, and then 5°C min^−1^ to 600°C, where the temperature was maintained for an additional 20 minutes, for a total run time of approximately 117 minutes. The products released from N117 thermolysis were absorbed using 0.5L 0.05mol L^−1^ NaOH solution and methanol. Afterwards, Nafion residues were immersed in these solutions and shaken for 20 min for an adequate products elusion. These absorption solutions were then filtered through 0.22μm membrane filter, and the filtrate was collected for subsequent analysis.

### LC/ESI-MS/MS analysis

HPLC was carried out on an Agilent Infinity 1260 series LC system (Agilent Technologies, Santa Clara, CA, USA). The system was equipped with G1379B degasser, G1312B binary pump, and G1329B autosampler. Chromatographic separation was performed at a flow rate of 300μL min^−1^ using a Thermo BDS Hypersil C_18_ column (2.1mm × 100mm, particle size 2.4μm) (Thermo Fisher Scientific, Waltham, MA) that was maintained at 30°C. The mobile phase consisted of 0.3% formic acid in water (A) and acetonitrile (B). The linear gradient was initially 90% A, which was held isocratic for 2 min, decreased to 10% in 9 min, which was held under this condition for 2.5 min, and returned back to the starting condition in 0.5 min followed by 8 min equilibration. The injection volume was 10μL, and elution time was 22 min for each sample.

Mass spectrometric analysis was performed with a Q-TOF MS (TripleTOF 5600, AB Sciex, Concord, ON, Canada) operating in the negative ion mode using an electrospray ion source. Mass range of TOF MS was *m/z* 50-1000. The other parameters were set as follows: curtain gas, 35 (arbitrary units); ion source gas 1, 55 (arbitrary units); ion source gas 2, 55 (arbitrary units); temperature, 550°C; ionspray voltage floating, -4500kV; declustering potential, -80V; collision energy, −10eV. The MS/MS experiments were conducted in product ion scan mode at variable collision energies (20–50eV). The data were acquired in a data-dependent mode that used criteria from the previous MS scan to select the target precursor ions that would be submitted to MS/MS fragmentation. The final chemical structure of an unknown compound was identified based on the accurate mass measurements of the parent ions and fragments obtained from the MS/MS experiments. The high-resolution LC-MS/MS data were acquired using Analyst TF 1.6 (AB Sciex) and processed using PeakView 1.2 (AB Sciex).

### Ion chromatography analysis (ICA)

F^−^ ions released from N117 thermolysis were studied using an ion-chromatography system (ICS-1000, Dionex, USA). This system was equipped with an autosampler (injection volume: 25μL), a pump, a degasser, a guard column, and a separation column (Dionex IonPac AS 12A, 4mm i.d × 200mm, USA) operating at 30°C. The mobile phase was 20mM KOH and flow rate was set at 1.0mL min^−1^. Data acquisition and processing were carried out using Chromeleon 6.80 software (Dionex Corporation, Sunnyvale, CA, USA).

## Supplementary Material

Supplementary Information Supplementary Information

## Figures and Tables

**Table 1:** Peak assignments of different thermolysis products of N117 in NaOH absorption solution.

Groups	Proposed structure	Observed m/z	Calculated m/z	Major MS/MS fragments (*m/z*)	*RT* (min)	Intensity (peak area)	*m/z* values of other analogues
1	CF_3_COO^−^	112.9870	112.9856	68.9953, 45.0005	0.863	1.77×10^6^	262.9738, 312.9721, 362.9674, 462.9630, 512.9618, 562.9593, 612.9575, 662.9552
	CF_3_(CF_2_)_6_COO^−^	412.9658	412.9664	368.9759, 218.9851, 168.9885	10.366	1.94×10^6^	
2	CF_3_(CF_2_)_5_CHFCOO^−^	394.9780	394.9758	330.9707, 180.9837, 118.9896	10.298	3.14×10^6^	294.9836, 344.9811, 444.9759, 494.9731, 594.9673, 644.9647, 694.9629, 744.9595
	CF_3_(CF_2_)_8_CHFCOO^−^	544.9698	544.9663	480.9682, 330.9783, 168.9884	11.145	7.94×10^5^	
3	CF_3_(CF_2_)_4_CF = CFCF_2_COO^−^	424.9921	424.9664	380.9779, 230.9847, 192.9869	8.362	4.35×10^6^	374.9818, 474.9754, 574.9961, 624.9673, 674.9550, 724.9635, 774.9514, 824.9579
	CF_3_(CF_2_)_4_CF = CF(CF_2_)_3_COO^−^	524.9636	524.9600	480.9780, 330.9844, 292.9862	9.110	5.75×10^5^	
4	CF_3_(CF_2_)_3_CF(COOH)COO^−^	338.9692	338.9721	230.9825, 208.9806, 180.9861	1.100	5.24×10^5^	438.9679, 488.9652, 538.9623, 588.9605, 638.9569, 688.9541, 738.9532, 788.9491
	CF_3_(CF_2_)_4_CF(COOH)COO^−^	388.9697	388.9689	344.9791, 280.9850, 258.9827	1.823	8.32×10^5^	
-	HSO_4_^−^	96.9624	96.9601	79.9598	0.863	1.33×10^6^	-
-	CHF_2_SO_3_^−^	130.9619	130.9620	79.9590	1.953	3.75×10^5^	-

**Table 2:** The proposed chemical structures of some representative *m/z* values detected in this study, and their possible cleavage sites (highlighted in red).

*m/z* values	Molecular formula	Proposed chemical structures
**412.9658**	C_8_F_15_O_2_^−^	
368.9759	C_7_F_15_	
218.9851	C_4_F_9_	
168.9885	C_3_F_7_	
**394.9780**	C_8_HF_14_O_2_^−^	
330.9707	C_7_F_13_	
180.9837	C_4_F_7_	
118.9896	C_2_F_5_	
**96.9624**	HSO_4_^−^	
79.9598	SO_3_	
**130.9619**	CHF_2_SO_3_^−^	
79.9590	SO_3_	

**Table 3:** Peak assignments of different thermolysis products of N117 in methanol absorption solution.

Groups	Proposed structure	Observed m/z	Calculated m/z	Major MS/MS fragments (*m/z*)	*RT* (min)	Intensity (peak area)	*m/z* values of other analogues
1	CF_3_COO^−^	112.9873	112.9856	68.9949, 44.9993	0.923	4.81×10^6^	312.9878, 362.9899, 462.9922, 512.9930, 662.9994, 713.0036
	CF_3_(CF_2_)_6_COO^−^	412.9717	412.9664	368.9806, 218.9876, 168.9904	10.362	8.42×10^5^	
2	CF_3_(CF_2_)_3_CHFCOO^−^	294.9905	294.9822	230.9909, 158.9882, 112.9850	11.109	8.08×10^6^	244.9953,344.9981, 394.9987, 495.0019, 595.0047, 645.0054, 695.0098, 745.0105
	CF_3_(CF_2_)_6_CHFCOO^−^	444.9944	444.9727	380.9920, 230.9915, 180.9902	10.286	7.96×10^5^	
3	CF_3_(CF_2_)_4_CF = CF(CF_2_)_2_COO^−^	474.9938	474.9632	430.9848, 280.9808, 230.9835	10.632	6.56×10^6^	224.9881, 274.9885, 374.9907, 424.9921, 524.9946, 624.9987, 675.0003, 725.0028
	CF_3_(CF_2_)_4_CF = CF(CF_2_)_4_COO^−^	574.9644	574.9568	530.9744, 380.9800, 330.9825	11.261	6.43×10^5^	
4	CF_3_CF = CFCF = CFCOO^−^	236.9847	236.9792	170.9888, 142.9928, 108.9902	0.980	5.24×10^5^	336.9906, 386.9922, 436.9935, 687.0254
	CF_3_CF = CFCF = CFCF_2_COO^−^	286.9848	286.9760	220.9881, 192.9917, 154.9932	9.979	8.32×10^5^	
5	CF_3_CF = CFCF_2_COOCO^−^	252.9848	252.9741	208.9844, 180.9592, 142.9922	11.006	9.09×10^5^	302.9853, 453.0097, 503.0107, 553.0125, 603.0129, 503.0109, 553.0125, 653.0149
	CF_3_CF = CF(CF_2_)_3_COOCO^−^	352.9721	352.9677	308.9998, 230.9866, 142.9924	10.486	9.61×10^5^	
6	CF_3_(CF_2_)_5_CHFCOOCF_2_CO^−^	472.9934	472.9676	394.9811, 308.9812, 242.9885	12.581	1.10×10^6^	373.0142, 573.0012, 623.0195, 673.0138
	CF_3_(CF_2_)_6_CHFCOOCF_2_CO^−^	522.9896	522.9644	444.9932, 428.9963, 358.9896	12.581	2.02×10^6^	
7	CF_3_(CF_2_)_3_CHFCOOCH(OH)O^−^	340.9925	340.9877	294.9863, 274.9785, 230.9881	11.120	8.23×10^5^	290.9849, 491.0097, 591.0127, 641.0146, 691.0177
	CF_3_(CF_2_)_4_CHFCOOCH(OH)O^−^	390.9877	390.9845	344.9841, 280.9861, 230.9982	11.657	2.20×10^6^	
8	CF_3_CF = CFCO^−^	158.9915	158.9875	92.9954	11.006	1.15×10^6^	258.9938, 409.0153, 659.0071, 709.0101, 959.0201
	CF_3_CF = CFCF_2_CO^−^	208.9867	208.9843	180.9911, 142.9937, 130.9936	11.863	6.98×10^5^	
-	HSO_4_^−^	96.9605	96.9601	79.9579	0.871	1.28×10^6^	-
-	CHF_2_SO_3_^−^	130.9619	130.9620	79.9584	1.895	6.49×10^5^	-
