# Supplementary material for: Characterization of the thermolysis products of Nafion membrane: A potential source of perfluorinated compounds in the environment
Source: Sci Rep. 2015 May 7;5:9859. doi: 10.1038/srep09859 (PMC5386195; doi:10.1038/srep09859)
Supplement: Supplementary Information [file srep09859-s1.pdf]

Supplementary Information

Characterization of the thermolysis products of Nafion membrane: A potential source of perfluorinated compounds in the environment

Mingbao Feng, Ruijuan Qu, Zhongbo Wei, Liansheng Wang, Ping Sun*, Zunyao Wang*

State Key Laboratory of Pollution Control and Resources Reuse, School of the Environment, Nanjing University, Nanjing 210023, P. R. China

*** Corresponding authors:**

Dr. Zunyao Wang, Fax: +86-25-89680358; E-mail: wangzy@nju.edu.cn.

Dr. Ping Sun, Fax: +86-25-83686580; E-mail: sunping@nju.edu.cn.

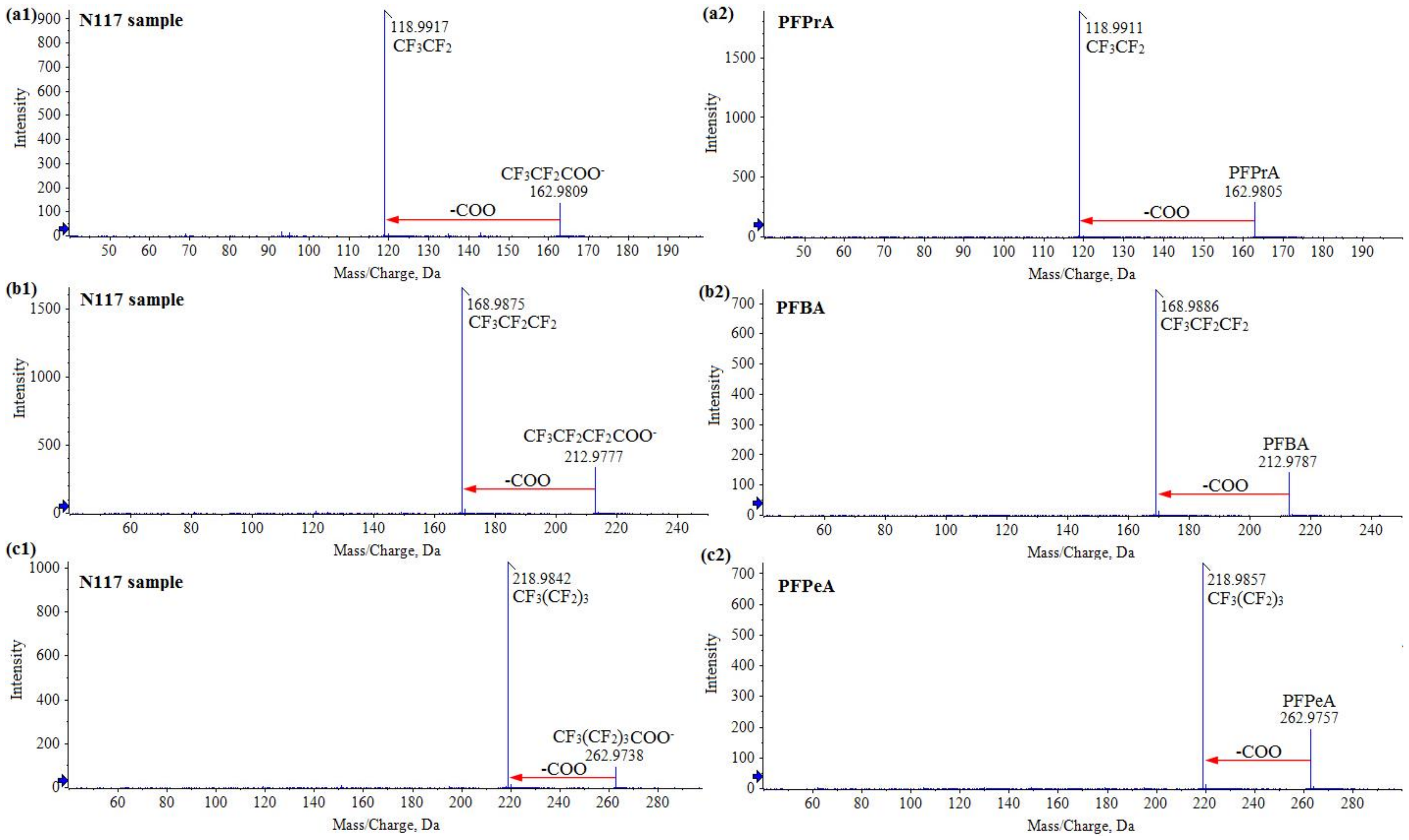

(a1)
N117 sample
118.9917
CF3CF2
CF3CF2COO-
162.9809
-COO
Intensity
Mass/Charge, Da
(a2)
PFPrA
118.9911
CF3CF2
PFPrA
162.9805
-COO
Intensity
Mass/Charge, Da
(b1)
N117 sample
168.9875
CF3CF2CF2
CF3CF2CF2COO-
212.9777
-COO
Intensity
Mass/Charge, Da
(b2)
PFBA
168.9886
CF3CF2CF2
PFBA
212.9787
-COO
Intensity
Mass/Charge, Da
(c1)
N117 sample
218.9842
CF3(CF2)3
CF3(CF2)3COO-
262.9738
-COO
Intensity
Mass/Charge, Da
(c2)
PFPeA
218.9857
CF3(CF2)3
PFPeA
262.9757
-COO
Intensity
Mass/Charge, Da

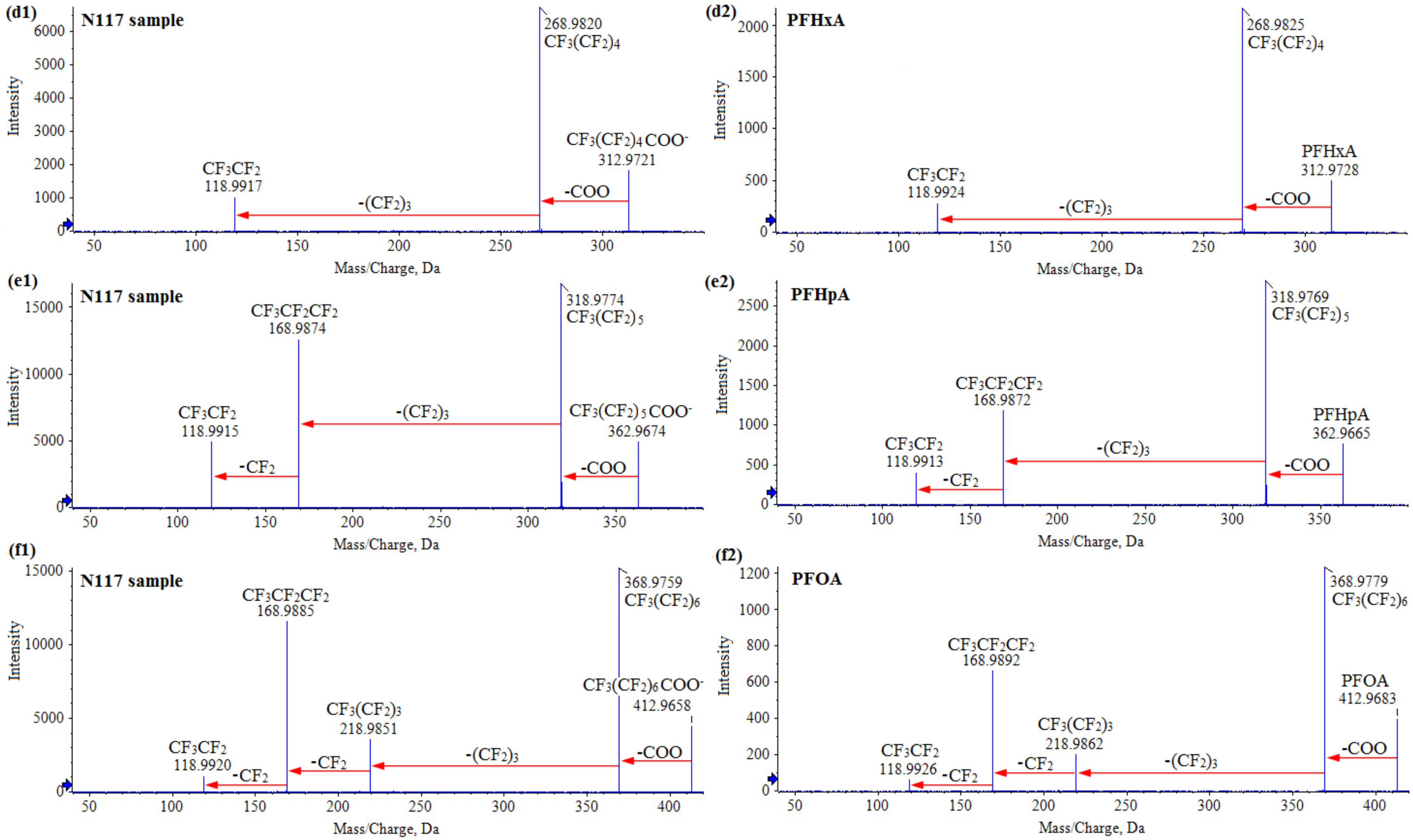
(d1)
N117 sample
268.9820
CF3(CF2)4
CF3(CF2)4COO-
312.9721
CF3CF2
118.9917
-(CF2)3
-COO
Intensity
Mass/Charge, Da
(d2)
PFHxA
268.9825
CF3(CF2)4
PFHxA
312.9728
CF3CF2
118.9924
-(CF2)3
-COO
(e1)
N117 sample
CF3CF2CF2
168.9874
318.9774
CF3(CF2)5
CF3(CF2)5COO-
362.9674
CF3CF2
118.9915
-(CF2)3
-CF2
-COO
(e2)
PFHpA
318.9769
CF3(CF2)5
CF3CF2CF2
168.9872
PFHpA
362.9665
CF3CF2
118.9913
-(CF2)3
-CF2
-COO
(f1)
N117 sample
368.9759
CF3(CF2)6
CF3CF2CF2
168.9885
CF3(CF2)6COO-
412.9658
CF3(CF2)3
218.9851
CF3CF2
118.9920
-CF2
-CF2
-(CF2)3
-COO
(f2)
PFOA
368.9779
CF3(CF2)6
CF3CF2CF2
168.9892
PFOA
412.9683
CF3(CF2)3
218.9862
CF3CF2
118.9926
-CF2
-CF2
-(CF2)3
-COO

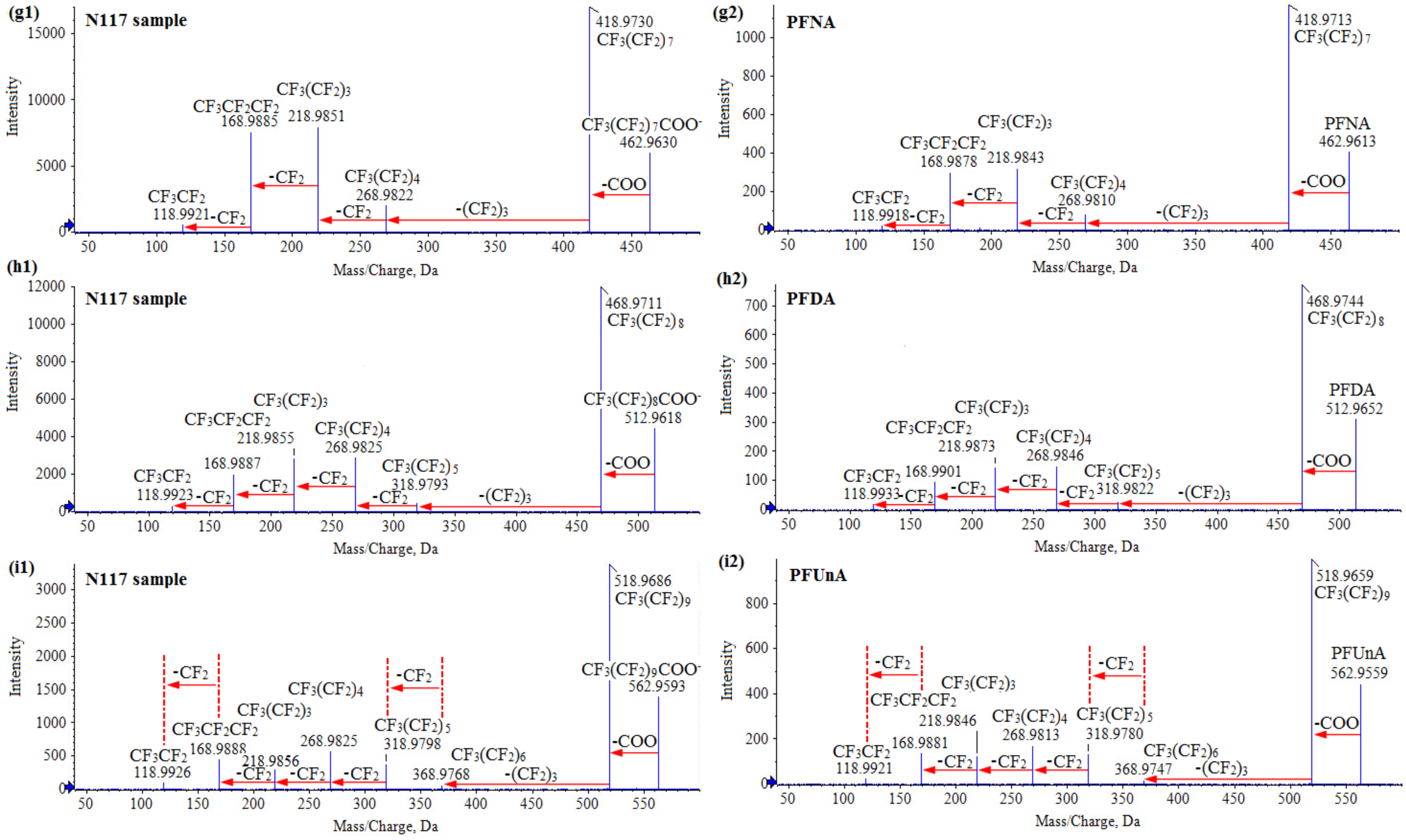

(g1)
N117 sample
418.9730
CF3(CF2)7
CF3(CF2)3
CF3CF2CF2
168.9885
218.9851
CF3(CF2)7COO-
462.9630
-CF2
-COO
CF3(CF2)4
268.9822
CF3CF2
118.9921
-CF2
-(CF2)3
Intensity
Mass/Charge, Da
(g2)
PFNA
418.9713
CF3(CF2)7
CF3(CF2)3
CF3CF2CF2
168.9878
218.9843
PFNA
462.9613
-CF2
-COO
CF3(CF2)4
268.9810
CF3CF2
118.9918
-(CF2)3
Intensity
Mass/Charge, Da
(h1)
N117 sample
468.9711
CF3(CF2)8
CF3(CF2)8COO-
512.9618
CF3(CF2)3
CF3CF2CF2
218.9855
CF3(CF2)4
268.9825
168.9887
-COO
CF3(CF2)5
318.9793
CF3CF2
118.9923
-CF2
-(CF2)3
Intensity
Mass/Charge, Da
(h2)
PFDA
468.9744
CF3(CF2)8
PFDA
512.9652
CF3(CF2)3
CF3CF2CF2
218.9873
CF3(CF2)4
268.9846
-COO
CF3(CF2)5
CF3CF2
168.9901
118.9933
318.9822
-CF2
-(CF2)3
Intensity
Mass/Charge, Da
(i1)
N117 sample
518.9686
CF3(CF2)9
CF3(CF2)9COO-
562.9593
-CF2
CF3(CF2)4
CF3(CF2)3
CF3CF2CF2
268.9825
CF3(CF2)5
318.9798
-COO
CF3CF2
168.9888
218.9856
CF3(CF2)6
118.9926
368.9768
-(CF2)3
Intensity
Mass/Charge, Da
(i2)
PFUnA
518.9659
CF3(CF2)9
PFUnA
562.9559
-CF2
CF3(CF2)3
CF3CF2CF2
218.9846
CF3(CF2)4
268.9813
CF3(CF2)5
318.9780
-COO
CF3CF2
168.9881
118.9921
CF3(CF2)6
368.9747
-(CF2)3
Intensity
Mass/Charge, Da

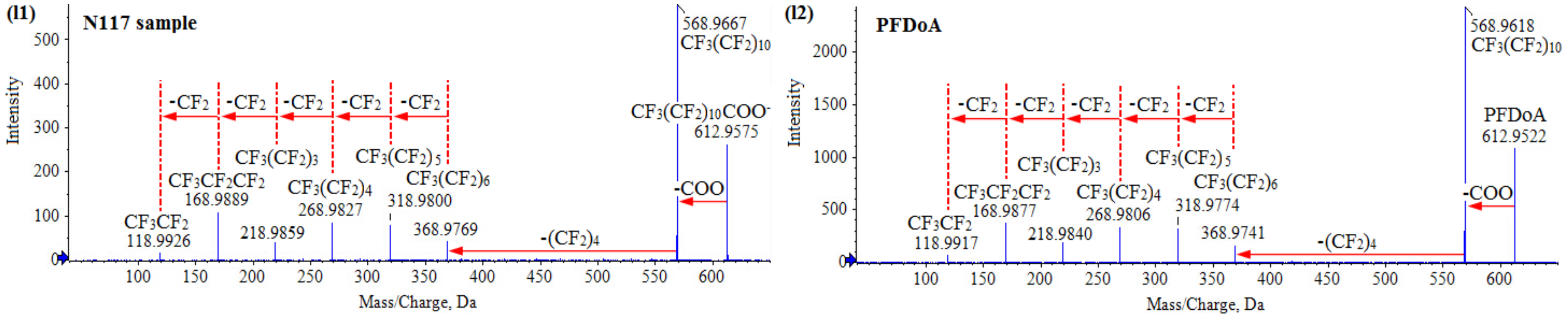

Supplementary Figure S1. Q-TOF MS/MS spectra of product ion scan of selected *m/z* values (a1, 162.9809; b1, 212.9777; c1, 262.9738; d1, 312.9721; e1, 362.9674; f1, 412.9658; g1, 462.9630; h1, 512.9618; i1, 562.9593; l1, 612.9575) of N117 thermolysis products, and the validations by the corresponding standard solutions of PFCAs (a2, PFPrA; b2, PFBA; c2, PFPeA; d2, PFHxA; e2, PFHpA; f2, PFOA; g2, PFNA; h2, PFDA; i2, PFUnA; l2, PFDoA; 50 μg L^{-1} for each compound) and the proposed structures for their major fragments.

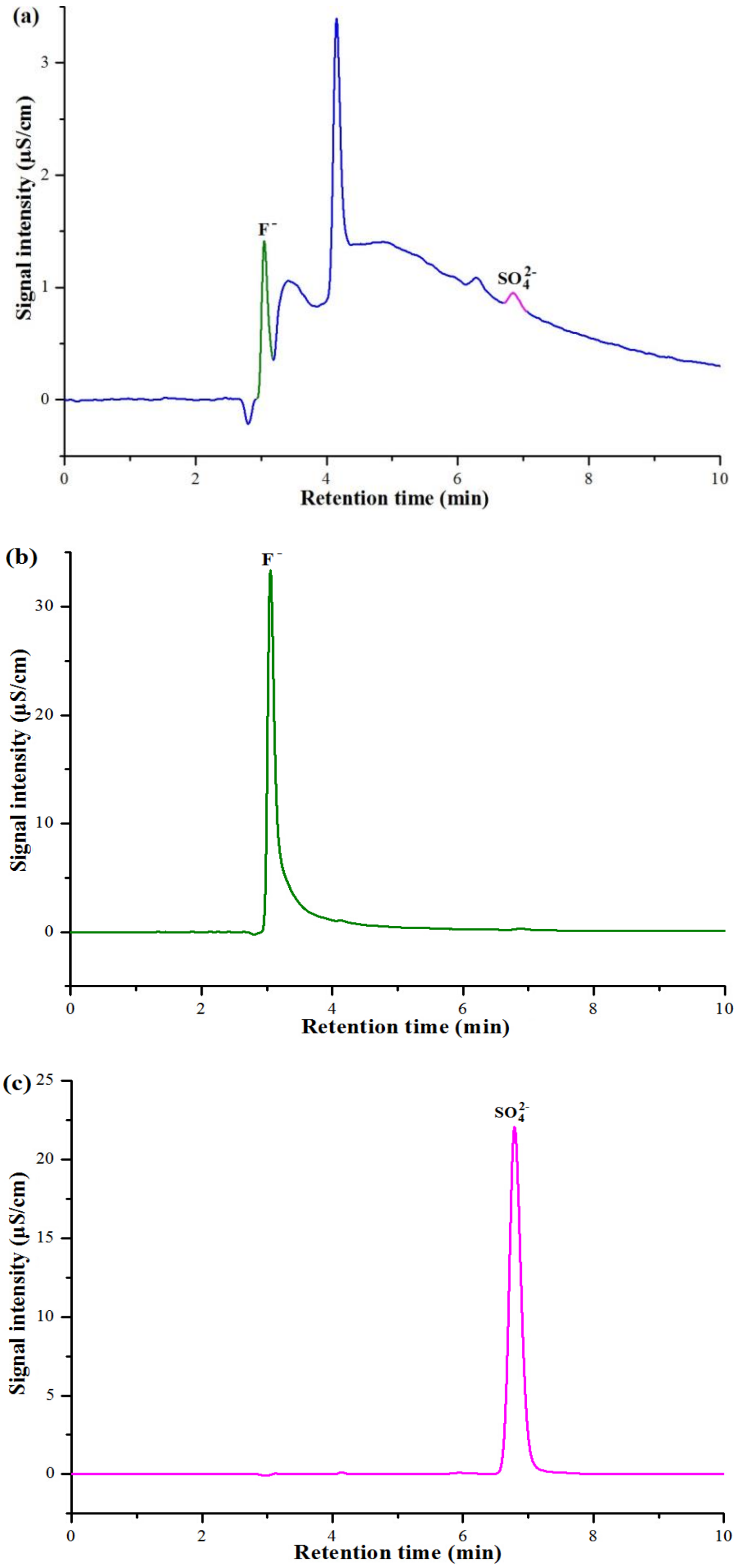

Supplementary Figure S2. The observed ion chromatogram (IC) of NaOH absorption solution (a), which indicates the formation of F^- and SO_4^{2-} ions during N117 thermolysis, NaF solution (20 mg L^{-1}; b) and Na_2SO_4 solution (20 mg L^{-1}; c).

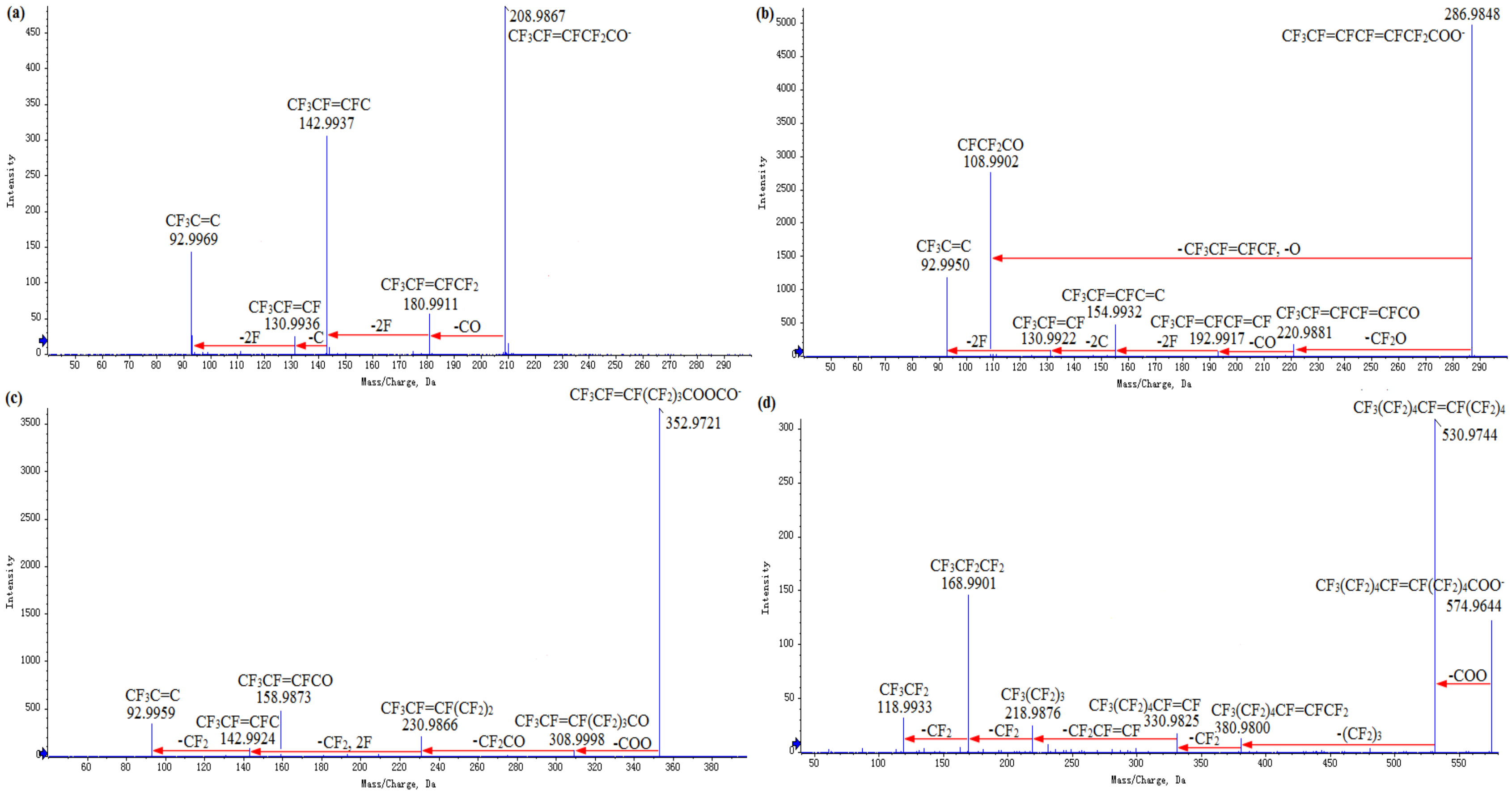

Supplementary Figure S3. Q-TOF MS/MS spectra of product ion scan of several representative *m/z* values of PFC analogues (a, 208.9867; b, 286.9848; c, 352.9721; d, 574.9644), and the proposed structures for their major fragments.

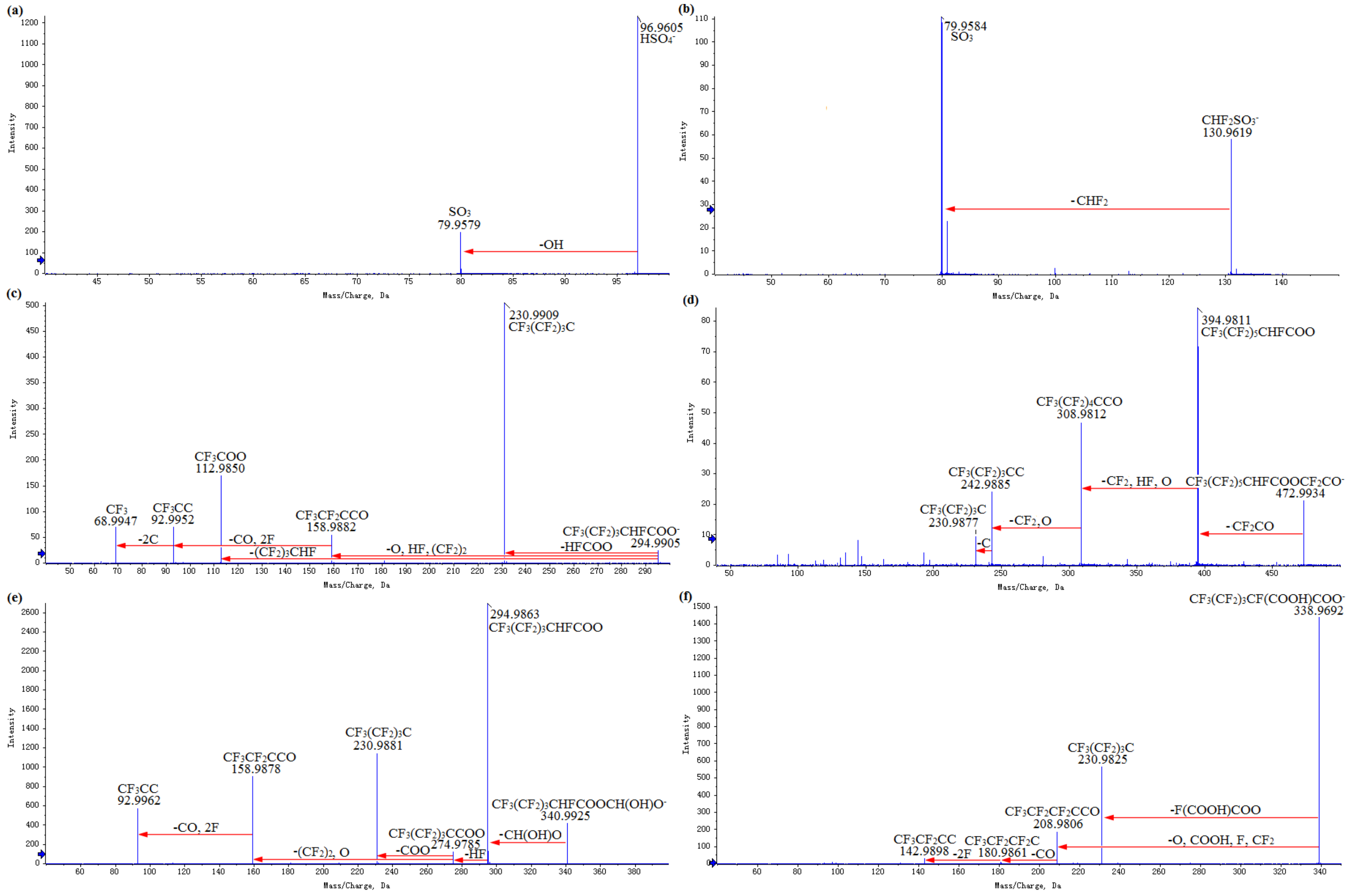
(a)
96.9605
HSO4-
SO3
79.9579
-OH
Intensity
Mass/Charge, Da
(b)
79.9584
SO3
CHF2SO3-
130.9619
-CHF2
(c)
230.9909
CF3(CF2)3C
CF3COO
112.9850
CF3
68.9947
CF3CC
92.9952
CF3CF2CCO
158.9882
-2C
-CO, 2F
-(CF2)3CHF
-O, HF, (CF2)2
CF3(CF2)3CHFCOO-
-HFCOO
294.9905
(d)
394.9811
CF3(CF2)5CHFCOO
CF3(CF2)4CCO
308.9812
CF3(CF2)3CC
242.9885
CF3(CF2)3C
230.9877
-CF2, HF, O
CF3(CF2)5CHFCOOCF2CO-
472.9934
-CF2,O
-CF2CO
-C
(e)
294.9863
CF3(CF2)3CHFCOO
CF3(CF2)3C
230.9881
CF3CF2CCO
158.9878
CF3CC
92.9962
-CO, 2F
-(CF2)2, O
CF3(CF2)3CCOO
274.9785
-COO
-HF
CF3(CF2)3CHFCOOCH(OH)O-
340.9925
-CH(OH)O
(f)
CF3(CF2)3CF(COOH)COO-
338.9692
CF3(CF2)3C
230.9825
-F(COOH)COO
CF3CF2CF2CCO
208.9806
-O, COOH, F, CF2
CF3CF2CC
142.9898
CF3CF2CF2C
180.9861
-2F
-CO

Supplementary Figure S4. Q-TOF MS/MS spectra of product ion scan of selected *m/z* values of several other products (a, 96.9605; b, 130.9619; c, 294.9905; d, 472.9934; e, 340.9925; and f, 338.9692), and the proposed structures for their major fragments.

Supplementary Table S1. The proposed chemical structures of several other *m/z* values observed in this study, and their possible cleavage sites (highlighted in red).

m/z values	Molecular formula	Proposed chemical structures
**208.9867**	$C_5F_7O^-$	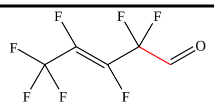
180.9911	C_4F_7	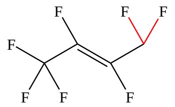
142.9937	C_4F_5	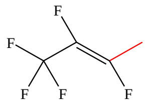
130.9936	C_3F_5	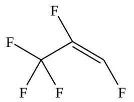
**352.9721**	$C_8F_{11}O_3^-$	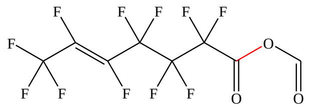
308.9998	$C_7F_{11}O$	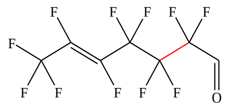
230.9866	C_5F_9	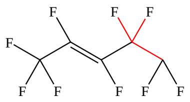
142.9924	C_4F_5	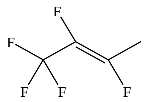
**574.9644**	$C_{12}F_{21}O_2^-$	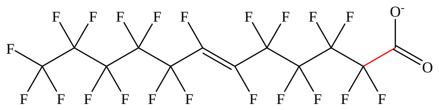
530.9744	$C_{11}F_{21}$	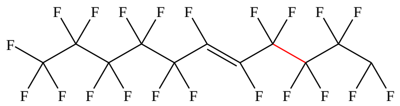
380.9800	C_8F_{15}	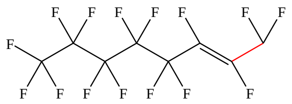
330.9825	C_7F_{13}	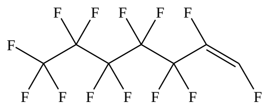

Supplementary Table S2. Chemical names, formula and molecular structures (linear isomers) of PFCA analogues ($C_nF_{2n+1}COOH$, n = 1-11) of interest in this study.

Chemical name	Formula	Abbreviation	Molar weight (g mol^{-1})	Chemical structure
Trifluoroacetic acid	CF_3COOH	TFA	113.99	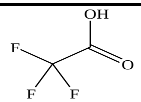
Perfluoropropanoic acid	C_2F_5COOH	PFPrA	163.99	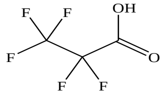
Perfluorobutanoic acid	C_3F_7COOH	PFBA	213.99	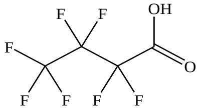
Perfluoropentanoic acid	C_4F_9COOH	PFPeA	263.98	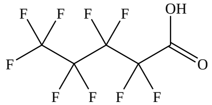
Perfluorohexanoic acid	$C_5F_{11}COOH$	PFHxA	313.98	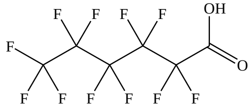
Perfluoroheptanoic acid	$C_6F_{13}COOH$	PFHpA	363.98	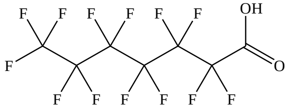

Perfluorooctanoic acid	$C_7F_{15}COOH$	PFOA	413.97	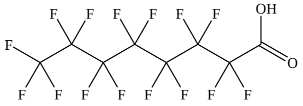
Perfluorononanoic acid	$C_8F_{17}COOH$	PFNA	463.97	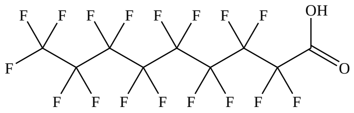
Perfluorodecanoic acid	$C_9F_{19}COOH$	PFDA	513.97	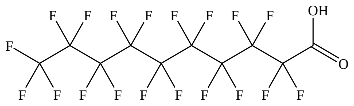
Perfluoroundecanoic acid	$C_{10}F_{21}COOH$	PFUnA	563.96	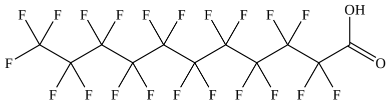
Perfluorododecanoic acid	$C_{11}F_{23}COOH$	PFDoA	613.96	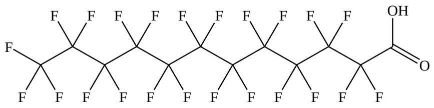

Supplementary Table S3. The thermal degradation products of perfluorosulfonic acid copolymer [a].

Compound	Evolution Temperature, °C	mg g^{-1} Sample
SO_2	280	15
CO_2	300	30
HF	400	- [b]
CO	400	3
R_fCOF	400	10 [c]
COF_2	400	3
COS	400	Trace
R_fOH	400	Trace

[a] Cited from DuPont Fuel Cells[1].

[b] Significant level but concentration could not be determined because HF reacts with and absorbs on cell walls.

[c] Mixture of products.

Supplementary text

Chemical degradation mechanism of Nafion in fuel cells. Currently, the unzipping reaction has been termed as a commonly accepted chemical degradation mechanism. It is generally initiated by the attack of some radicals on the H-containing end groups, causing the release of HF and CO_2 and the formation of new carboxylate groups at the chain ends, and finally resulting in the membrane degradation[2,3]. The proposed reactions between $OH^{\bullet}$ and end groups $-CF_2COOH$ are shown below (eq 1-3)[2,4].

$$R_F\text{-}CF_2COOH + OH^{\bullet} \rightarrow R_F\text{-}CF_2^{\bullet} + CO_2 + H_2O \quad (1)$$

$$R_F\text{-}CF_2^{\bullet} + OH^{\bullet} \rightarrow R_F\text{-}CF_2OH \rightarrow R_F\text{-}COF + HF \quad (2)$$

$$R_F\text{-}COF + H_2O \rightarrow R_F\text{-}COOH + HF \quad (3)$$

Supplementary References

1. DuPont Fuel Cells. Safe Handling and Use of Perfluorosulfonic Acid Products (Technical Information). (2009).
2. Wu, J. F. *et al.* A review of PEM fuel cell durability: Degradation mechanisms and mitigation strategies. *J. Power Sources* **184**, 104-119 (2008).
3. Danilczuk, M., Lancucki, L., Schlick, S., Hamrock, S. J. & Haugen, G. M. In-depth profiling of degradation processes in a fuel cell: 2D spectral-spatial FTIR spectra of Nafion membranes. *ACS Macro Lett.* **1**, 280-285 (2012).
4. Curtin, D. E., Lousenberg, R. D., Henry, T. J., Tangeman, P. C. & Tisack, M. E. Advanced materials for improved PEMFC performance and life. *J. Power Sources* **131**, 41-48 (2004).
